# Metabolic–Epigenetic Axis in Pluripotent State Transitions

**DOI:** 10.3390/epigenomes3030013

**Published:** 2019-07-31

**Authors:** Cristina D’Aniello, Federica Cermola, Eduardo J. Patriarca, Gabriella Minchiotti

**Affiliations:** Stem Cell Fate Laboratory, Institute of Genetics and Biophysics “Adriano Buzzati-Traverso”, CNR, 80131 Naples, Italy

**Keywords:** pluripotent stem cell transitions, metabolites availability, DNA and histone methylation, histone acetylation

## Abstract

Cell state transition (CST) occurs during embryo development and in adult life in response to different stimuli and is associated with extensive epigenetic remodeling. Beyond growth factors and signaling pathways, increasing evidence point to a crucial role of metabolic signals in this process. Indeed, since several epigenetic enzymes are sensitive to availability of specific metabolites, fluctuations in their levels may induce the epigenetic changes associated with CST. Here we analyze how fluctuations in metabolites availability influence DNA/chromatin modifications associated with pluripotent stem cell (PSC) transitions. We discuss current studies and focus on the effects of metabolites in the context of naïve to primed transition, PSC differentiation and reprogramming of somatic cells to induced pluripotent stem cells (iPSCs), analyzing their mechanism of action and the causal correlation between metabolites availability and epigenetic alteration.

## 1. Introduction

Cell fate decisions result in the acquisition of a new identity, defined by specific molecular and phenotypic features. Cell state transitions (CST) are finely regulated at multiple levels, underlying fundamental biological processes, both during development and in the progression of several diseases. In some cases, CST results in the acquisition of fate, which is permanent (e.g., stem cell/lineage specification during embryogenesis), whereas in others, the transition is reversible and therefore highlights the plastic behavior of cells (e.g., the epithelial to mesenchymal transition). Signaling pathways and gene cascades activation are among the most well-defined and studied molecular mechanisms involved in CST; however, many other aspects can influence the decision of a cell to undergo a different fate, including variations in the concentration/level of metabolites [1]. The metabolism of a cell results from a complex network of inputs, including growth factors, nutrients, and oxygen availability. It has been proposed that metabolic changes may either be a consequence of CST or may be instructive; specifically, metabolic reprogramming itself may create a permissive context to induce a phenotype transition [1,2]. Pluripotent stem cells (PSCs) show unique metabolic features in terms of energy consumption, metabolite flux and macromolecule synthesis. In this context, changes in the availability of different nutrients have been reported to influence the dynamic equilibrium between different cellular states, such as the balance between self-renewal/proliferation and induction of differentiation, the exit from the naïve state towards the primed state of pluripotency, and the acquisition of a pluripotent identity during the reprogramming of somatic cells to induced pluripotent stem cells (iPSCs) [3,4]. PSCs usually are bivalent in their energy production, i.e., produce energy/ATP from both oxidative phosphorylation (OX/PHOS) and glycolysis, although it strictly depends on the pluripotent state in which they reside. Indeed, as mouse embryonic stem cells (mESCs) exit the naïve state and become primed, their energy production switches from OX/PHOS to glycolysis [5]. Then, as differentiation occurs, cells produce energy through OX/PHOS again [6]. Human embryonic stem cells (hESCs), which are similar to primed Epiblast stem cells (EpiSCs), seem to display high levels of glycolytic activity [6,7]. Of note, during human PSCs (hPSCs) lineage specification, while definitive endoderm and mesoderm undergo a metabolic switch, nascent ectoderm maintains a high glycolytic flux [8]. In contrast, when somatic cells lose their identity and acquire pluripotency during reprogramming, they undergo different metabolic states acquiring at the end a glycolytic metabolism, which is the typical energy metabolism of primed cells [9,10,11]. Metabolism has been tightly linked to cell fate decision, both in pluripotent and adult stem cells, as it may impact on key gene-regulatory networks or influence the epigenetic landscape of cells [12,13]. The epigenome is indeed sensitive to the levels of several intermediary metabolites as these are the substrates, cofactors or products of chromatin-modifying enzymes [2]. For instance, histone acetylation relies on acetyl-CoA abundance, whereas DNA and histone methylation rely on *S*-adenosylmethionine (SAM) levels. Moreover, JmjC domain-containing (JMJ) histone demethylases and Ten Eleven Translocation (TET) DNA demethylases engage ketoglutarate (αKG) as a substrate and vitamin C (ascorbic acid, VitC) as a cofactor, and are inhibited by fumarate, succinate and 2-hydroxyglutarate. Of note, the availability of these metabolites and many other macro- and micro- nutrients, such as amino acids, and vitamins, may affect histone and DNA modification both globally and at specific sites, resulting in alteration of gene expression. However, although emerging studies support the link between the spatio-temporal control of metabolite concentration and the epigenetic alterations, the causal link between these two, as well as the mechanisms underlying the specificity in the metabolic regulation of chromatin modifications and gene expression, are still unresolved questions [14].

In summary, changes in the abundance of different metabolites could modify the epigenome in a reversible manner, thus accounting, at least in part, for the plastic behavior of cells. Here we review how the metabolic shift associated with cell fate decision in PSCs results in the abundance of specific intermediary metabolites, which render chromatin-modifying enzymes sensitive. We discuss the mechanism by which different metabolites may influence the epigenome, and whether other biological processes may be influenced in response to fluctuations of their availability. We also discuss the effect(s) of specific metabolites in the context of CST, either by modulating exogenously their availability (addition or deprivation) or by genetically manipulating the expression of the enzymes involved in their biosynthesis. Finally, we examine if the metabolic–epigenetic crosstalk results in specific transcriptome signatures, which underlie the new cellular identity acquired.

## 2. *Acetyl Coenzyme* A

**Figure 1:**
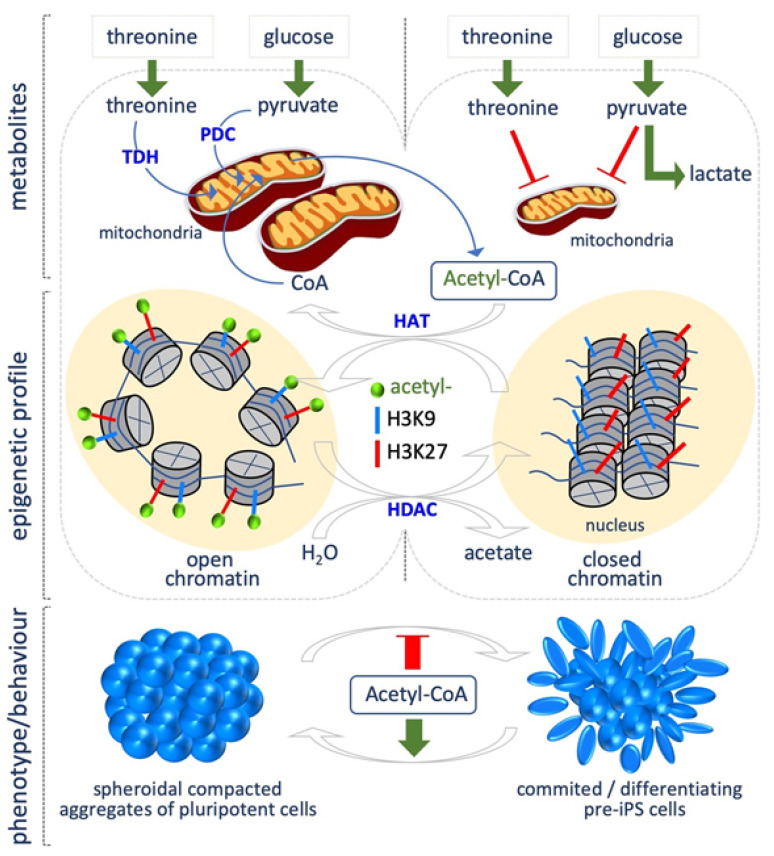
Acetyl-CoA abundance maintains pluripotent stem cell identity. In the undifferentiated state, acetyl-CoA is generated from threonine catabolism, through the threonine dehydrogenase (TDH), and from glycolysis-derived pyruvate, which is converted into acetyl-CoA by the pyruvate dehydrogenase complex (PDC). In the differentiated state TDH is downregulated, and glycolysis-derived pyruvate is preferentially converted into lactate. Acetyl-CoA abundance impacts on histone residues acetylation, e.g., H3K9 and H3K27, promoting the transition from a committed/differentiated to a pluripotent state. Red T bar: repression; green arrow: induction (bottom).

Fluctuations in *acetyl coenzyme* A (acetyl-CoA) levels, in response to nutrient availability or metabolic reprogramming, determine the global levels of histone acetylation, which in turn modulate transcriptional responses [14]. Intracellular acetyl-CoA concentration directly depends on primary metabolic processes, such as break down of carbohydrates, fatty acids and amino acids, thus being an important indicator of the metabolic state of the cells during growth and development [15,16]. Acetyl-CoA is produced in mammals in different cellular compartments, such as the mitochondria, cytosol and nucleus. Mitochondrial acetyl-CoA can be synthetized through different pathways. First, glycolysis-derived pyruvate can be converted into acetyl-CoA by the pyruvate dehydrogenase complex (PDC). Second, when cells are starved, β-oxidation is activated, resulting in fatty acids breaking down and the production of acyl-CoAs, which are transported into the mitochondria to produce acetyl-CoA. Acetyl-CoA can also derive from amino acid or ketone body metabolism. Finally, it can be synthetized from acetate by the acyl-CoA Synthetase short-chain family member 1 (ACSS1). Acetyl-CoA produced in mitochondria reacts with oxaloacetate to produce citrate, which can be oxidized in the tricarboxilic acid cycle (TCA) to produce ATP, or can exit the mitochondria for the synthesis of cytosolic acetyl-CoA through the ATP citrate lyase (ACLY). Cytosolic acetyl-CoA is used for fatty acid biosynthesis and histone acetylation (Figure 1). Acetyl-CoA can be also synthetized in the cytosol and in the nucleus, using as precursor exogenous acetate through the acyl-CoA Synthetase short-chain family member 2 (ACSS2).

A close relationship between acetyl-CoA levels and histone acetylation has been described during cell state transitions. Indeed, acetyl-CoA abundance and thus histone acetylation, in particular of histone H3, is crucial for maintaining an open chromatin state, and thus the epigenetic plasticity, which is a key feature of PSCs.

Yanes et al. showed that ESCs are characterized by high levels of highly unsaturated fatty acids, which decrease during differentiation [17]. Accordingly, a recent study highlights the critical role of fatty acid synthesis for maintaining PSC identity also during the process of cellular reprogramming [18]. Through magnetic resonance (NMR), Moussaieff et al. showed that the early phases of hESC differentiation are accompanied by a decrease of glycolysis-derived acetyl-CoA. Indeed, PSCs produce high levels of both acetyl-CoA and acetate, thus suggesting that the pyruvate-acetyl-CoA step, rather than the classical glycolysis versus OX/PHOS metabolism, is crucial to preserve pluripotency [19]. Accordingly, acetate is accumulated during somatic cell reprogramming, concomitantly with the acquisition of a pluripotent identity [9], and, of note, acetate supplementation (10 mM) maintains both mouse and human embryonic stem cells (mESCs, hESCs) in the naïve state, and preserve H3K9/27 acetylation, which is required to maintain an open chromatin state, typical of PSCs [20]. This finding supports a crucial role for glycolysis-derived acetyl-CoA in modulating the balance between pluripotency and differentiation (Figure 1). Mali et al. showed that butyrate, a naturally occurring short-chain fatty acid, improved the efficiency of iPSCs generation from both adult and fetal fibroblasts. Butyrate is a well-known histone deacetylase (HDAC) inhibitor [21]. The authors found that a transient butyrate treatment (0.25–0.5 mM) increased histone H3K9 acetylation, DNA demethylation of pluripotency-associated genes, including *DPPA2*. Forced expression of *DPPA2* recapitulates the effects of butyrate treatment [22].

Another source of acetyl-CoA is the metabolism of amino acids, including threonine. Indeed, threonine dehydrogenase (Tdh) activity converts threonine into acetyl-CoA and glycine (Figure 1). In line with the above described role of histone acetylation in maintaining pluripotency, *Tdh* gene is much more expressed (about 200 times) in ESCs than in murine embryonic fibroblasts (MEF) [23].

## 3. Nicotinamide Adenine Dinucleotide

Nicotinamide adenine dinucleotide (NAD) is an essential cofactor for several enzymes involved in a multiplicity of cellular responses. Among them, NAD is required by Sirtuins, a class of histone deacetylase (HDAC) that removes acetyl-groups from histones. Upon this reaction, acetyl-groups are transferred to ADP-ribose moiety of NAD. Nicotinamide adenine dinucleotide exists in an oxidized (NAD^+^) and reduced (NADH) form, and can be synthesized either through the *de novo* pathway, using tryptophan as a metabolic precursor, or through the *salvage* pathway, assembling together nicotinamide (NAM), nicotinic acid (NA) and nicotinamide riboside to give rise to NAD^+^. In mammals, calorie restriction increases NAD^+^ levels, while a hyper-caloric diet reduces NAD^+^ levels [24].

The family of Sirtuins, which contains seven members in mammals, have been largely described as key regulators in development and stem cell biology [25]. In particular, they are crucial regulators of stem cell maintenance and differentiation through several mechanisms, including protection from ROS and apoptosis, and epigenetic modification of specific targets. Sirtuin 1 is a key regulator of mouse and human ESCs. Indeed, it is down regulated during hESC differentiation, leading to epigenetic activation of developmental genes [26]. In addition, it modulates mESC differentiation [27] and preserves naïve pluripotency by deacetylating *Oct4* and preventing the induction of *Otx2* gene [28]. Furthermore, Sirtuin 1 preserves ESC identity by antagonizing Dnmt3l (DNA Methyltransferase 3 Like) and protecting against excessive DNA methylation [29]. Sirtuin 6 increases the efficiency of cellular reprogramming [30], and preserves ESC pluripotency preventing spontaneous differentiation toward neuroectoderm [31].

It is thus reasonable to assume that fluctuations of NAD^+^ levels may influence the epigenetic landscape of PSC; however, few studies report the effect of NAD abundance on PSC identity/differentiation, and the correlation between NAD^+^ levels and epigenetic modification has not been investigated so far. For instance, by using a NAD^+^ biosynthesis inhibitor, Son et al. investigated the role of NAD^+^ availability in somatic cell reprogramming, and found that NAD^+^ depletion affects the efficiency of iPSCs. Supplementation of the NAD precursor NAM (1 mM) replenished NAD^+^ levels and increases reprogramming efficiency. Mechanistically, the authors suggest that this is due to the pro-survival role of NAD^+^, which protects cells from apoptosis and senescence by reducing the oxidative stress. However, the effect of NAD^+^ abundance on histone acetylation levels was not investigated [32]. Recently, Meng et al. reported that Nicotinamide (5–10 mM) promotes hPSCs survival and differentiation, and suggest that it occurs through the inhibition of ROCK kinase [33]. Meanwhile, Kropp et al. showed that inhibition of NAD^+^ salvage pathway is toxic for hPSCs in culture [34]. Furthermore, the NAD precursor NAM (10 mM) has been reported to facilitate PSC differentiation toward different lineages, including neural, pancreatic, and cardiac [35,36,37,38,39,40].

All these studies point to a crucial role for NAD^+^ in the maintenance of pluripotency and regulation of differentiation. Given the well-documented role of Sirtuins in these biological processes, analyzing the effect of NAD^+^ or NAM on histone acetylation levels may offer new molecular mechanism underlying NAD^+^-dependent modulation of CST.

## 4. Folate Coenzyme

**Figure 2:**
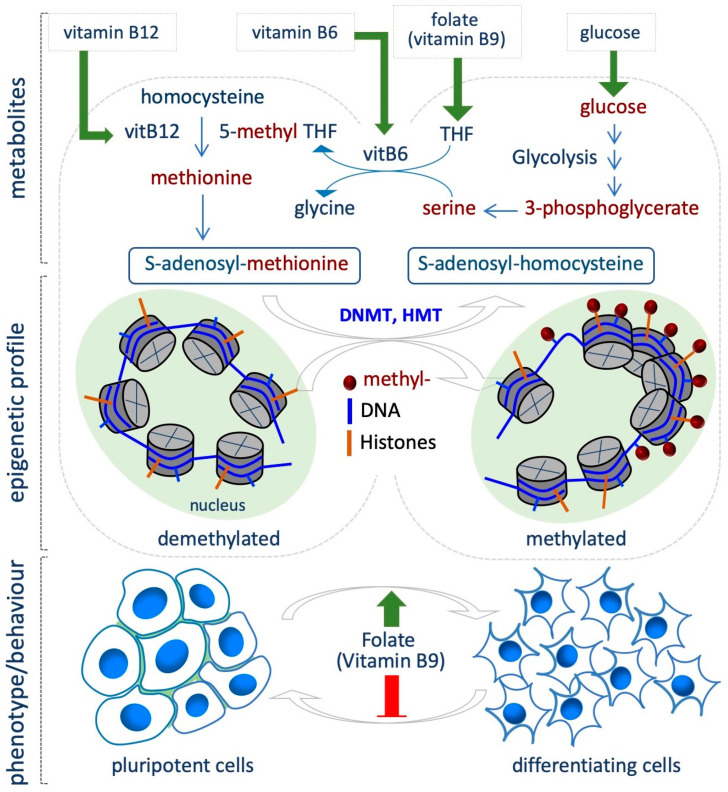
One-carbon metabolism-dependent control of DNA and histone methylation regulates the balance between pluripotent stem cell self-renewal and differentiation. Several micronutrients and metabolites influence the generation of s-adenosylmethionine (SAM), the universal donor of methyl groups for DNA and histone methyltransferases (DNMT and HMT). Folate (vitamin B9) is introduced by diet and converted to tetrahydrofolate (THF), and then to 5-methylTHF, which donate a methyl group to homocysteine to generate methionine. Serine, derived from glycolysis, provides a methyl group for the generation of 5-methyl-THF from THF. Vitamins B6 and B12 are essential for these reactions. While folate deficiency maintains and induces the pluripotent state, folate abundance promotes exit from the pluripotent state and induction of differentiation. Red T bar: repression; green arrow: induction (bottom).

The availability of folate (vitamin B9) is directly linked to DNA and histone methylation levels, since folate is involved in the one-carbon metabolism, which provides methyl groups for the methylation reactions. One-carbon metabolism involves two cycles, i.e., folate and methionine cycle, to utilize one-carbon units from a variety of nutrients (mostly glucose and amino acids) for nucleotide and lipid synthesis, for the control of the redox status and for methylation reactions. Once introduced by diet, Folic acid is first converted to dihydrofolate and then to tetrahydrofolate (THF). As THF, it enters the folate cycle where the methylenetetrahydrofolate reductase (MTHFR) converts methylene-THF into 5-methyl-THF, and then, the 5-methyltetrahydrofolate-homocysteine methyltransferase (MTR) transfers a methyl group from 5-methyl-THF to homocysteine to generate methionine. Methionine adenosyltransferase (MAT) catalyzes the synthesis of *S*-adenosylmethionine (SAM), which is used as universal donor of methyl groups by the DNA- and histone- methyltransferases (DNMT and HMTs). *S*-adenosylhomocysteine (SAH) is generated as a product of this reaction, and is further hydrolyzed to homocysteine to close the cycle (Figure 2). Vitamins B2, B6 and B12 are essential for SAM biosynthesis, since involved in the conversion of THF in 5-methyl-THF (B2 and B6), and homocysteine into methionine (B12) [24,41].

Evidence from several sources indicates the involvement of folate in modulating the epigenetic landscape in different contexts of cell transitions. For instance, Chandrasekaran et al. integrating time-course metabolomic profiles and a computational model of metabolism, revealed significant differences between naïve and primed mouse PSCs. In particular, one-carbon metabolism emerged as a key pathway that differs between the two states, being much more active in the primed state. Indeed, the primed state show increased sensitivity to knockout of reactions in folate/SAM/one-carbon metabolism, while the naïve state is more sensitive to reactions in the oxidative phosphorylation (OX/PHOS) and tricarboxilic acid (TCA) cycle [12].

Accordingly, several studies link folate levels and stem cell transition and differentiation by modulating methylation both globally and at specific sites. It is well known that high levels of DNA methylation act as a barrier during the reprogramming of somatic cells to iPSCs. Interestingly, folic acid deficiency promotes demethylation of *Oct4* and *Nanog* promoters in MEFs, resulting in increased reprogramming efficiency (Figure 2) [42]. In this context, Hu and colleagues identified small-molecules compounds that improved the efficiency of iPSC generation under folic acid deprivation; however the impact of folic acid deprivation on epigenetic modification was not investigated [43].

It has been postulated that ESCs can be used to test the effect of folic acid deficiency on self-renewal and pluripotency features through DNA methylation [44]. Indeed, Chang et al. demonstrated a direct correlation between the Long Interspersed Nucleotide Element-1 (LINE-1), which exerts a key role in the maintenance of genome structure and function during embryonic development, and folic acid deficiency in ESCs (0.5–4 mg/L) [45]. Specifically, the authors tested different degrees of folic acid restriction in ESCs, and found that it gradually induces hypomethylation of LINE-1, without altering mESCs proliferation and differentiation [45]. However, other apparently contradictory studies showed that folic acid maintains the naïve pluripotent state under CHIR99021 (GSK3 inhibitor, Wnt agonist) culture conditions, and promotes somatic cell reprogramming [46]. Furthermore, folic acid-deficient ESCs fail to proliferate, accumulating in G0/G1 and undergo apoptosis. The effect of folic acid deprivation on DNA methylation levels was not investigated [47].

Folate metabolism is also important in the self-renewal/proliferation and differentiation potential of the neural stem cells (NSCs) in vitro and in vivo (Figure 2). Specifically, its deficiency determines neural tube defects during development, pointing to a key role of folate in neural progenitor differentiation [48]. Folate deficiency effects have also been characterized in in vitro models of neural differentiation. In particular, folic acid-dependent DNA methylation has been characterized in the neural rosettes (NR), 3D tubular structures that emerge from hESC differentiation, and that recapitulate the events occurring during neural tube development. The authors identified NR-specific enhancer elements, and showed that folic acid-associated DNA methylation change (CpGs) occurs at the NR regulatory elements close to genes required for neural tube formation and metabolism [49]. Accordingly, folic acid depletion affects NR formation from rhesus monkey ESCs [50]. A recent study also supports the role of folic acid administration in improving neural differentiation, and specifically investigated the effect of folic acid exposure in a model of iPSCs derived from fetuses with neural tube defects. Of note, folic acid treatment improved the proper formation and differentiation of neural tube structures, with concomitant expression of specific markers [51]. However, although these studies highlight a crucial role for folate in modulating cell fate decision, i.e., neural differentiation, the direct link between folate availability and epigenetic changes requires further investigation.

## 5. Amino Acids

Amino acid metabolism has been reported to influence cell fate decision, in particular, the levels of several amino acids are critical for the maintenance of PSC identity and behavior [52].

### 5.1. Threonine

Among the essential amino acids, threonine has been reported to influence the epigenetic landscape through its catabolism. Threonine is catabolized in the mitochondria where threonine Dehydrogenase (TDH) enzyme generates glycine and acetyl-CoA. While acetyl-CoA feeds the tricarboxylic acid cycle (TCA), glycine, through the Glycine cleavage system, enters the one-carbon metabolism, generating 5,10-Methylene-THF, providing methyl groups for the generation of *S*-adenosylmethionine (SAM), the universal donor for the methylation reactions. This process is critical for the maintenance of PSC identity and plasticity [53,54]. Indeed, threonine catabolism is dynamically regulated in mESCs, in which the *Tdh* gene is highly induced, reflecting the reduced levels of threonine in undifferentiated ESCs compared to differentiated embryoid bodies (EBs). Besides threonine, the level of other metabolites changes during ESC differentiation, defining distinct metabolic profiles of the two cellular states, e.g., acetyl-CoA and folic acid are less abundant in differentiating EBs, while methyl-tetrahydrofolate level is high [53]. Wang et al. showed that specific deprivation of threonine from the culture media slows down ESC growth rate and strongly reduced alkaline phosphatase (AP) levels, in a dose-dependent manner (ranging from 0 to 400 µM) This effect was mainly explained by reduced DNA synthesis, with threonine catabolism-derived products also being involved in purine synthesis [53].

Ng Shyh-Chang et al. found significant metabolic changes when analyzing the reprogramming from somatic cells to iPSCs, with iPSCs being similar to ESCs. During the reprogramming of MEFs to iPSCs, threonine and folate decrease, while SAM increases. Therefore, the metabolic enzymes that channel threonine into SAM, such as Tdh, are more abundant in mESCs than in MEF [23]. Accordingly, induction of *Tdh* transcription, and thus of threonine catabolism, enhances reprogramming efficiency, being a positive regulator of the process [55]. Besides purine synthesis, one-carbon metabolism fuels methyl groups for the methylation reactions. Indeed, by tracing the fate of [U-^13^C]threonine Ng Shyh-Chang et al. found that threonine is converted into acetyl-CoA and glycine by mESCs, and [U-^13^C]threonine-derived glycine donates its methyl group to generate 5-methyltetrahydrofolate and SAM. Accordingly, short time depletion of threonine from the culture medium of mESCs reduced SAM levels, which in turn affects specifically di- and tri-methylation of histone H3 lysine 4 (H3K4me3), which has an essential role in the maintenance of pluripotency. Surprisingly, the authors did not find changes in the acetylation levels of histone H3, which is also crucial for the maintenance of an open chromatin state in PSCs (Figure 1). Deprivation of threonine also impairs mESC growth and increased differentiation. However, both the reduction of cell growth and H3K4me3 can be reverted by supplementation of threonine or glycine together with pyruvate, supporting a contribution of pyruvate-derived acetyl-CoA in the maintenance of mESC identity. Finally, the authors confirmed the effect of threonine catabolism on histone methylation and hence on ESC identity and pluripotency by depleting *Tdh* [23]. A global hypo-methylated state of histone and DNA is generally associated with the naïve pluripotent state. However, trimethylation of H3K4, which is generally associated with active transcription, is crucial to maintain pluripotency. Of note, among other histone methyltransferases (HMTs), the H3K4me3 writer Set1A has the highest Km for SAM, meaning that requires high levels of SAM for its activity [56], and supporting the requirement of threonine-derived SAM for ESC pluripotency.

### 5.2. Methionine/S-Adenosylmethionine

Methionine is an essential amino acid, important for *S*-adenosylmethionine (SAM) synthesis through the methionine-adenosyltransferase (MAT). SAM is the methyl donor of the histones and DNA methyltransferases, and its level depends on the availability of methionine introduced by diet. Upon the methylation reaction, SAM is converted to *S*-adenosylhomocysteine (SAH) and then to homocysteine by SAH hydrolase. Finally, homocysteine is converted again to methionine by 5-methyltetrahydrofolate-homocysteine methyltransferase (MTR).

Human ESCs/iPSCs do not rely on threonine catabolism, since they have a non-functional *TDH* pseudogene, but keep methionine and SAM levels constant and equilibrated to maintain their undifferentiated state. Indeed, Shiraki and colleagues showed that hESCs/hiPSCs actively convert *S*-methyl-5′-thioadenosine (MTA), a by-product of polyamine biosynthesis, into methionine and then SAM. Accordingly, methionine deprivation impairs hESCs/hiPSCs survival and self-renewal/pluripotency and poises them for differentiation. While short methionine deprivation is reversible, if prolonged it results in growth arrest and cell death. The authors showed that methionine deprivation leads to SAM levels reduction and a concomitant decrease of H3K4me3 levels and global DNA methylation levels, which are rescued by SAM supplementation. According to reduction of cell growth, loss of pluripotent identity and induction of differentiation, p53–p38 signaling is induced and *NANOG* expression is suppressed upon methionine deprivation. In line with these findings, knockdown of *MAT2A* and *MAT2B* genes, which are involved in SAM synthesis from methionine, decreases hPSC self-renewal, which is rescued by supplementation with methionine or SAM (100 µM) [57]. Similarly to that observed for threonine catabolism in mESCs, this study points to a crucial role for SAM levels in hPSCs, through methionine metabolism, which is important to keep H3K4me3 mark, and thus maintain the undifferentiated state. Yet, the mechanism by which SAM availability specifically influences H3K4 methylation, but not other histone residues, is still unknown.

Recent data point to a key role of one-carbon metabolism in somatic cell reprogramming. For instance, it has been found that during reprogramming iPSCs activate the methylation cycle and maintain an optimal *S*-adenosylmethionine (SAM)/*S*-adenosylhomocysteine (SAH) ratio. This step is crucial to prevent the increase of homocysteine levels, which might alter the global DNA methylation level, and provide a pool of methyl groups required to maintain the proper methylation levels for the stem cell state [58]. Recently, it has been shown that methionine metabolism is modulated by the NAD^+^-dependent protein deacetylase Sirt1 both in mESCs and in the early phases of embryo development [59]. Specifically, Sirt1 regulates SAM synthesis by controlling the expression of *Mat2a*, whose promoter is directly controlled by the Sirt1 deacetylase substrate Myc. In the absence of Sirt1, mESC colonies show a flat phenotype with reduced levels of Alkaline Phosphatase (AP), a marker of pluripotency. Moreover, the conversion of methionine to SAM is impaired. Accordingly, the effects of methionine deprivation are more accentuated on *Sirt1* KO ESCs with reduction of histone methylation. However, *Mat2a* overexpression rescues H3K4me3, *Nanog* expression and blocks differentiation [59].

Complementary to these findings, Sperber et al. investigated how the metabolic signatures regulate the epigenetic profiles associated with the naïve to primed pluripotency transition. Interestingly, they observed that induction of naïve features in hESCs [60,61,62,63,64] is accompanied with metabolic changes similar to that observed for the mouse counterpart. Indeed, primed hESCs show lower oxidative phosphorylation (OX/PHOS) compared to naïve hESCs, as well as increased levels of fructose (1,6/2,6-) bisphosphate and lactate, suggesting active glycolysis in the primed state. Moreover, other metabolites are abundant in the primed state, including methionine, nicotinamide, long-carbon chain lipids and SAM. In contrast, in the naïve state, the fatty acid β-oxidation is increased. Most interestingly, the level of 1-methylnicotinamide (1-MNA), a product of Nicotinamide N-methyltransferase (NNMT), is upregulated in the naïve state [65]. The authors showed that the levels of NNMT, a SAM consuming enzyme, are dynamically regulated during the naïve to primed transition. Indeed, NNMT correlates with the naïve state, leading to reduction of SAM and of the repressive histone marks H3K27me3 and H3K9me3. In contrast, NNMT reduction in the primed state corresponds to increased SAM levels, which are thus available for histone methylation. Accordingly, forced expression of NNMT delays naïve to primed transition, while SAM supplementation (500 µM) induces primed metabolic profile in naïve hESCs. The epigenetic alterations due to NNMT levels determine repression of the Wnt pathway and activate the hypoxia-inducible factor (HIF) [65].

Thus, methionine/SAM influence the epigenetic profile with different effects, depending on the pluripotent state and the cellular context, i.e., PSC differentiation or naïve to primed transition. H3K27me3 generally increases during the transition from naïve to primed. Moreover, in PSCs, H3K4me3 and H3K27me3 are associated with genes that may be either poised for activation or repression, the so called the bivalent domains [66]. This may explain different requirements of SAM in the pluripotent vs primed state. All together these studies support the idea that a complex balance between different inputs, such as the activity of methylating and demethylating enzymes, finely controls cell identity.

### 5.3. Proline

The non-essential amino acid proline is synthetized from glutamate, which derives from glutamine. In a first step, Aldh18a1 enzyme catalyzes the reduction of glutamate in Pyrroline-5-carboxylate (P5C), then P5C is converted to proline by P5C Reductase (Pycr1). Ornithine, a precursor of proline, can be converted in P5C in the mitochondria. Proline is catabolized in the mitochondria through the activity of Prodh enzyme, which converts proline to P5C, which in turn can be converted again to proline in the cytoplasm, thus generating a Proline-P5C cycle.

**Figure 3:**
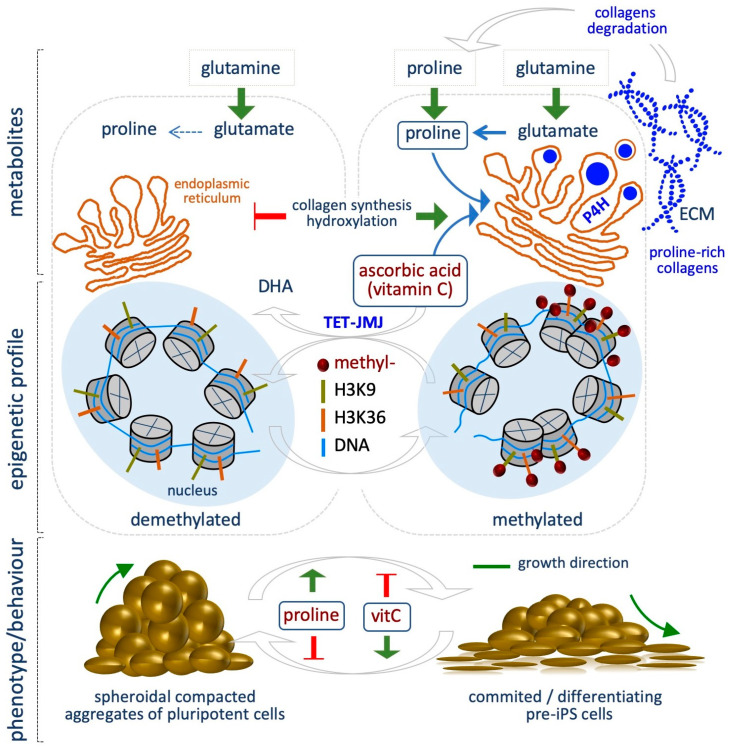
Proline availability influences pluripotent stem cell identity and behavior. Pluripotent stem cells show a specific intrinsic starvation of proline. Exogenously provided proline leads to increased collagen synthesis and hydroxylation in the endoplasmic reticulum (ER), where prolyl-4 hydroxylases (P4h) consume vitamin C (VitC). Consequently, VitC availability in the nucleus decreased, resulting in reduced TET and JMJ activity, and increased DNA and histone methylation. This metabolic perturbation is associated with pluripotent stem cell exit from the naïve state and induction of an early-primed state of pluripotency. VitC antagonizes proline effects and promotes a naïve state. Red T bar: repression; green arrow: induction (bottom).

Emerging evidence indicate that l-Proline (l-Pro) influences the epigenetic landscape of mESCs, regulating histones and DNA methylation levels [67,68]. Indeed, l-Pro availability modulates mESC plasticity, being a key determinant of ESC identity and behavior (Figure 3) [68,69,70]. Several lines of evidence support this conclusion. First, mESC identity relies on a specific intrinsic shortage of l-Pro, whose levels are kept under the control of the amino acid starvation pathway (AAR/Atf4) [71]. Indeed, Atf4, the effector of the AAR pathway, controls the expression of l-Pro biosynthesis enzymes *Aldh18a1* and *Pycr1*. As l-Pro levels increase (e.g., by exogenously provided l-Pro, 150–500 µM), l-Pro-tRNA is loaded and protein synthesis increased. This eventually results in increased ESC proliferation, exit from the naïve state and a reversible phenotypic and molecular transition. Specifically, upon exogenous l-Pro supplementation, ESCs are forced to acquire an early-primed state of pluripotency, defined by transcriptome, metabolic and epigenetic features that resemble the pre-primed state of Epiblast-like cells (EpiLCs). Indeed, while mESCs are bivalent in their energy production, l-Pro-induced cells (PiCs) undergo a metabolic reprogramming, switching to a glycolytic metabolism, which is typical of the primed state [72]. Additionally, PiCs acquire mesenchymal/motile and invasive features similar to that of epithelial to mesenchymal transition (EMT), leading to the conclusion that l-Pro induces an embryonic stem cell to mesenchymal-like transition (esMT) [67]. Interestingly, esMT is fully reversible either upon l-Pro withdrawal or vitamin C (VitC) supplementation, which in turn promotes the reversed mesenchymal-like to embryonic stem cell transition (MesT). Remarkably, VitC, but not other antioxidant (e.g., NAC and GSH), antagonizes the process (Figure 3) [67].

l-Pro-induced phenotypic transition is associated with a global and genome-wide increase of histone and DNA methylation levels (Figure 3). Specifically, l-Pro supplementation increases the global levels of H3K9me3/me2 and H3K36me3. ChIP-Seq showed that H3K9 methylation is altered at 1,6621 sites, while H3K36me3 at 8648 sites in l-Pro-treated cells, with the highest increase at noncoding regions of the genome. A significant fraction (27%) of the 1521 l-Pro-deregulated genes, in particular the ones associated with the acquisition of the mesenchymal features, show a change of the H3K9 methylation status [67].

l-Pro supplementation also modifies DNA methylation levels in ESCs. Specifically, it increases the global levels of 5-methylcytosine (5mC) while reducing 5-hydroxymethylcytosine (5hmC), as quantified by liquid chromatography followed by mass spectrometry (LC–MS) [68]. Of particular relevance, VitC antagonizes l-Pro-dependent increase of both histone and DNA methylation. Reduced representation bisulfite sequencing (RRBS) analysis identified about 1000 differentially methylated regions (DMRs) in l-Pro- and VitC-treated ESCs. The large majority of these DMRs (≥ 90%) are oppositely modified by l-Pro and VitC availability [68]. Accordingly, the DNA methylation profiles of l-Pro-treated cells and *Tet* triple knock out (TKO) ESCs are highly overlapping, highlighting that l-Pro and VitC availability impacts on a common mechanism of action [73].

How fluctuation in the levels of the metabolites analyzed so far, namely acetyl-CoA, NADH, vitamin B9, methionine/SAM, impacts on the epigenetic landscape of cells may be explained by the fact that, apart from l-Pro, they are substrates or cofactors for the epigenetic enzymes, which are sensitive to their availability. The mechanism underlying l-Pro- induced epigenetic modification has been recently explored [73]. Indeed, it has been proposed that increased l-Pro availability boosted collagen synthesis and hydroxylation, which is catalyzed in the endoplasmic reticulum (ER) by a group of VitC/αKG/Fe^+2^-dependent dioxygenases, namely Prolyl-hydroxylase (P4h). This process consumes VitC in the ER, decreasing the availability of this co-factor/enhancer in the nucleus for the activity of another group of VitC/αKG/Fe^+2^-dependent dioxygenases, i.e., the JumonjiC-domain containing histone demethylases (JmjC) and the Ten-eleven Translocation (Tet) DNA demethylases. A sudden increase of collagen hydroxylation (e.g., upon l-Pro supplementation) thus generates a competition for VitC availability between P4h and the epigenetic enzymes, resulting in increased histone and DNA methylation levels (Figure 3) [73].

We speculate that l-Pro availability may influence stem cell plasticity and behavior during development, e.g., when tissue remodeling increases extracellular matrix (ECM)/ collagen degradation, producing high levels of free l-Pro, which may act as a signal for CST. Moreover, it has been reported that *Aldh18a1* gene is induced in the Primitive Endoderm, exactly when the pluripotent cells are specified in the inner cell mass (ICM) of the blastocyst [74]. This aspect is of great interest and deserves further investigations.

## 6. Alpha-Ketoglutarate

Alpha-ketoglutarate (αKG) is a substrate of VitC/Fe^+2^-dependent dioxygenases superfamily, which includes the DNA demethylases (Tet) and the histone demethylases (Jmj), involved in multiple biological processes. Tet and Jmj utilize αKG and oxygen to hydroxylate and remove methyl groups on cytosines and lysines on DNA and histones, respectively. During the reaction, αKG is oxidized to succinate, Fe^+2^ is oxidized to Fe^+3^, and carbon dioxide (CO_2_) is produced.

αKG is a key molecule in the Krebs cycle (citric acid cycle or tricarboxylic acid cycle, TCA), where it is produced by oxidative decarboxylation of isocitrate by isocitrate dehydrogenase (Idh). The TCA cycle results in the production of NADH, which is fed into the oxidative phosphorylation (electron transport) pathway to produce energy in the form of ATP. Alternatively, some amino acids, namely proline, arginine, lysine, and glutamine, can be converted into glutamate, which in turn undergoes oxidative deamination by glutamate dehydrogenase and produces αKG.

**Figure 4:**
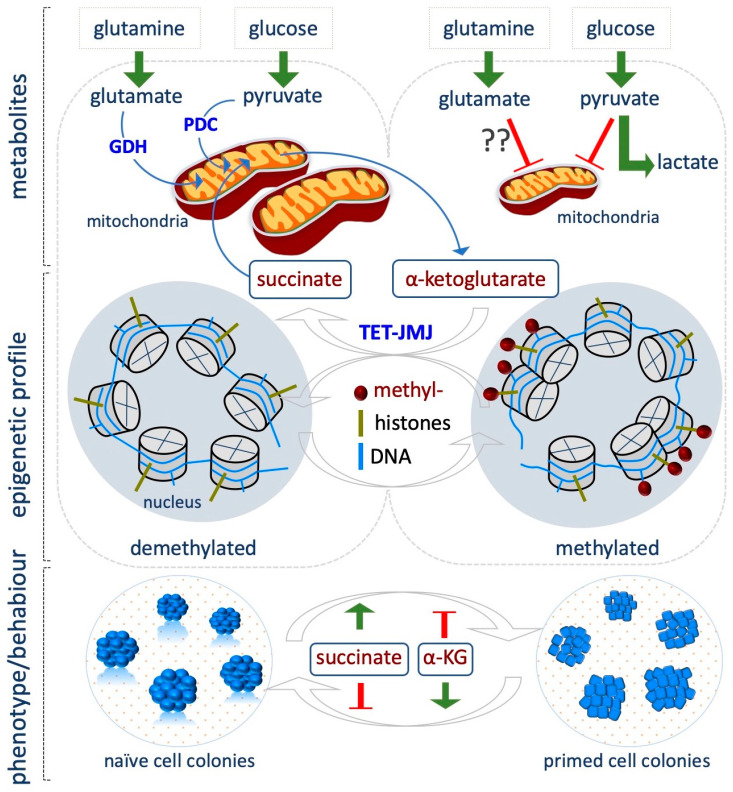
Alpha-ketoglutarate (αKG)/ succinate ratio modulates the naïve to primed transition. αKG abundance in the naïve state depends on glutamate, through the glutamate dehydrogenase (GDH), and on glucose-derived pyruvate. Pyruvate is converted in acetyl-coA by the pyruvate dehydrogenase complex (PDC), and enters the Krebs cycle in the mitochondria, generating citrate, isocitrate and then αKG. High levels of αKG, a key cofactor of the DNA and histone demethylases, TET and JMJ, guarantee DNA and histone demethylation, promoting a naïve state in mouse ESCs. Succinate, which is produced from αKG oxidation, pushes cells towards differentiation. Red T bar: repression; green arrow: induction (bottom).

Being a key player in the demethylation reactions, αKG is actively involved in the modulation of the epigenetic landscape associated with CSTs and a critical role of αKG is emerging in preserving mESCs pluripotency [75]. According to their bivalent energy metabolism, naïve ESCs use both glutamine and glucose to maintain a high αKG/succinate ratio compared to the differentiated cells (Figure 4) [75]. High levels of αKG guarantee the optimal activity of Tet and Jmj enzymes, which results in a global hypomethylated epigenome that is a feature of naïve cells. Indeed, when ESCs are grown in glutamine-free medium, the global levels of several histone lysine residues, including H3K9me3/H3K27me3/H3K36me3 and H4K20me3 increase. However, supplementation of dimethyl-αKG (DM-αKG, 4 mM), a cell-permeable form of αKG, reverses histone methylation, thus proving a causal link exists between αKG availability and the epigenetic modifications observed. Remarkably, DM-αKG supplementation reduces global DNA methylation, and increases expression of Tet target genes as well as of several markers of inner cell mass (ICM) and germline cells. This effect is abrogated in *Tet1*/*2* double knock out (KO) ESCs, supporting the critical role of αKG as cofactor for Tet activity. αKG-dependent reduction of histone and DNA methylation and the concomitant increase in the expression of key pluripotent markers well correlate with the improved generation of domed-shaped tridimensional alkaline phosphate positive (AP^+^) ESC colonies. In contrast, supplementation of the cell-permeable dimethyl-succinate (4 mM) pushes ESCs towards differentiation, thus providing a strong indication that αKG/succinate ratio regulates stem cell identity, at least in part by modulating the epigenome, although chromatin independent effects cannot be ruled out (Figure 4) [75].

Accordingly, Hwang et al. demonstrated that αKG levels drop gradually as mESC differentiation proceeds, and treatment with αKG delays differentiation keeping a high percentage of AP^+^ colonies [76]. Interestingly, they found that phosphoserine aminotransferase 1 (Psat1), which is a target of Oct4/Sox2/Nanog complex, is critical in determining mESC self-renewal and pluripotency by regulating the levels of αKG, which in turn modulates DNA and histone methylation [76]. Indeed, in the absence of *Psat1*, αKG levels decrease, leading to a reduction of 5hmC and an increase of H3K9me3 and H3K36me3 global levels, with H3K9me3 enriched at promoters of core transcription factors, which results in downregulation of *Oct4* and *Nanog* expression and ESC differentiation. Of note, this effect is rescued by DM-αKG supplementation (0.5–1 mM).

Recently, Tischler et al. investigated how metabolic changes influence the specification of primordial germ cells (PGCs), which occurs during a narrow time window of competence that is acquired as ESCs exit from the naïve state and differentiate towards the epiblast-like cells (EpiLCs) state. Single cell RNASeq confirmed that the metabolic pathways are dynamically modulated during the transition from naïve to primed, with OX/PHOS and αKG levels being predominant in the naïve state. In line with a role of αKG in maintaining the naïve state, supplementation of DM-αKG (4 mM), and at a lesser extent citrate, a TCA metabolite upstream of αKG, impairs EpiLCs induction, retaining high level of the pluripotency marker Rex1. In contrast, DM-succinate treatment (4 mM) reduces Rex1 levels [77]. The authors suggest that this effect may be mediated by epigenetic changes. This highlights the concept that the metabolic switch during the naïve to primed transition is important for the acquisition of the developmental competence for the PGC fate. Accordingly, *Idh2* is highly expressed in PGCs and αKG supplementation improves PGC induction. Remarkably, αKG also extends the competent time window of EpiLCs for PGC differentiation by maintaining longer a chromatin modification signature characteristic of naïve pluripotency. Indeed, αKG supplementation of EpiLCs antagonizes H3K9me2 accumulation at enhancers of naïve genes, while H3K27me3 levels increase at these loci [77].

In a recent study, it has been suggested that the impact of αKG/succinate ratio on self-renewal or differentiation depends on both pluripotent state and the cellular context. Indeed, in hPSCs, the αKG/succinate ratio influences their identity in the opposite way, as compared to mESCs [78]. hPSCs, which exhibit transcriptome, metabolic and epigenetic features that resemble that of primed EpiSCs, show reduced oxidative phosphorylation (OX/PHOS) as compared to their differentiated counterparts, suggesting that TCA cycle intermediates production may be reduced. However, although hPSCs have low OX/PHOS, they utilize both [U-^13^C]glucose and [U-^13^C]glutamine to fuel TCA and produce high levels of αKG. In contrast, their early-differentiated counterparts only utilize glutamate [78]. In contrast to that observed in mESCs, the addition of DM-αKG (4–12 mM) on primed hPSCs and mouse EpiSCs accelerates their differentiation towards different lineages, such as neuroectoderm and endoderm. In contrast, DM-succinate supplementation (16 mM) or inhibition of Succinate Dehydrogenase A (SDHA), which converts succinate into fumarate, delays differentiation and maintains high levels of the pluripotency markers SSEA4 and OCT4. Decreasing αKG levels with different inhibitors also delays differentiation. Accordingly with the role of αKG as a substrate of TET and JMJ enzymes, its supplementation increases the levels of DNA 5-hydroxymethylcitosyne (5hmC) and reduces methylation levels of different histone residues (H3K4me3, H3K427me3, H3K9me3 and H3K36me3) [78].

These processes have been recently investigated in vivo, and the role of αKG in developing embryo has been reported [79]. Specifically, the treatment with αKG improves the blastocyst rate, the number of inner cell mass (ICM) and the fetal growth after embryo transfer, and this occurs, at least in part by modulating the activity of Tet and increasing the 5hmC/5mC ratio [79].

## 7. Vitamin C

Besides its well known role as an antioxidant, ascorbic acid [vitamin C (VitC)] is a cofactor/ enhancer of the αKG-dependent dioxygenases and thus controls critical cellular processes. VitC guarantees the continuous catalytic activity of these enzymes, since it serves for the reduction of ferric to ferrous (Fe^3+^ to Fe^2+^), which is produced at each oxidation/hydroxylation cycle. Indeed, although the first hydroxylation reaction can be performed in the absence of VitC, the reaction cannot proceed if Fe^3+^ is not oxidized back to Fe^2+^. Upon conversion of Fe^3+^ to Fe^2+^, the oxidized form of VitC, termed dehydroascorbic acid, is formed and can be rapidly reduced back to VitC [68]. Thus, VitC is essential for the Tet –dependent hydroxylation of 5-methylcitosine (5mC) residues to 5-hydroxymethylcitosine (5hmC) as well as for histone demethylation catalyzed by the Jmj demethylases [80].

In most mammals, VitC can be synthetized from glucose via the enzymatic action of L-gulono-γ-lactone oxidase (GULO). However, humans lack a functional *GULO* gene and thus cannot synthetize VitC, which must be introduced by diet and absorbed through the sodium-dependent VitC transporters.

Given its crucial role in promoting DNA and histone hypomethylation, the effect of VitC availability has been tested in a significant number of studies focused on maintenance and establishment of the pluripotent identity. Seminal studies revealed that VitC treatment (1–200 µg /mL) enhances the efficiency of both mouse and human iPSC generation, in particular improving the transition from pre-iPSCs to iPSCs [81,82]. Of note, neither NAC nor vitamin E was able to promote cell reprogramming, thus ruling out the possibility that the effect was due to VitC antioxidant activity. Esteban et al. suggested that VitC might enhance the reprogramming efficiency by modulating the activity of the epigenetic enzymes [81]. Indeed, histone and DNA methylation are considered as a barrier for reprogramming. Accordingly, VitC (50 µg/mL) enhances the activity of Jhdm1a/1b, the histone demethylases of H3K36me2/me3 [83], and reduces the levels of H3K9me3, regulating its status at the promoter of pluripotency loci, and improving the transition of pre-iPSCs to iPSCs [84]; these findings provided the first evidence connecting histone demethylation and VitC-induced reprogramming. These studies were thus further corroborated and extended. For instance, it has been shown that VitC supplementation (50 µg/mL) prevents aberrant hypermethylation of the imprinted *Dlk1-Dio3* cluster, thus allowing generation of “good” fully reprogrammed iPSCs, i.e., iPSCs showing all the characteristics of pluripotent ESCs, including the ability to generate adult mice by tetraploid complementation assay [85]. Complementary to these findings, it was shown that the germ-cell marker *Dppa3*, which is expressed in “high-grade” and “low-grade” chimera production competent iPSCs but not in pre-iPSCs, is activated during reprogramming by VitC (50 µg/mL) [86]. In line with these findings, it has been shown that VitC induces the expression of different ESC–specific microRNAs (miRNAs), including *miRNA290-295*, *miRNA17-92* clusters, and the miRNAs of the *Dlk1-Dio3* imprinting region by demethylating their promoters [87].

In addition, VitC is essential for Tet-dependent DNA demethylation that induces activation of pluripotency genes during reprogramming (0.01–50 µg/mL) [88,89]. Gao et al. showed that Tet1 activity facilitates reprogramming by promoting *Oct4* demethylation and activation together with other important pluripotency genes [90].

VitC, but not other antioxidants, promotes Tet activity increasing 5hmC levels in mESCs. This effect is suppressed in *Tet1* and *2* double knockout ESCs. Of note, VitC (100 µg/mL) induces demethylation of the regions that normally gain methylation in vivo after embryo implantation [91]. Moreover, VitC supplementation (500 µM) antagonizes l-Pro-dependent increase of histone and DNA methylation, maintaining a hypomethylated epigenome and pushing the cells towards a naïve-like state [67,68] (Figure 3). Similarly, Gao et al. showed that VitC preserves mESC colony morphology and prevents differentiation by down-regulating different differentiation -specific genes and up regulating the expression of pluripotency factors. In particular, *Nanog* protein levels increased upon VitC treatment [92]. In hESCs, VitC (50 µg/mL) induces DNA demethylation of bivalent genes, which is a fundamental step in somatic cell reprogramming [93]. VitC (50 µg/mL), as enhancer of TET activity, together with retinoic acid (vitamin A), which induces TET2 and 3 expression, synergistically cooperate to reduce 5mC levels and maintaining a naïve pluripotent state [94]. However, the use of culture conditions that promote naïve pluripotency, including VitC supplementation, with the consequent loss of DNA methylation, may lead to karyotype instability and the activation of transposons [95].

VitC availability also influences stem cell differentiation, although the mechanism in not completely understood, and most likely relies on Tet-dependent DNA demethylation. For instance, *Tet1–3* triple knockout affects ESC differentiation [96], and *TET2* deficiency affects mesoderm and hematopoietic differentiation in hESCs. Of note, methylation of the *NANOG* promoter correlates with the absence of *TET2* [97]; conversely, *TET2* overexpression correlates with *NANOG* promoter demethylation/hypomethylation and maintenance of ESCs in an undifferentiated state, i.e., with a block in ESC differentiation [98]. Finally, *Tet1* and *2* knockout ESCs show developmental defects when injected in mouse blastocyst [99], and *Tet1*-depleted ESCs form aggressive teratomas in immunocompromised mice, which are mainly composed by endoderm, and trophoblastic giant cells [100]. Of note, *Tet1* and *Tet3* deletion increases transcriptome variability during early embryogenesis [101,102].

VitC supplementation improves iPSC differentiation towards the cardiac lineage (10^−4^ mol/L) [103,104] enhancing cardiac progenitor cell specification and increasing proliferation via MEK-ERK1/2 pathway by promoting collagen synthesis (0.2–250 µg/mL) [105]. Several studies also support the role of VitC availability in promoting the differentiation of PSCs towards connective tissues including osteoblast [106] and osteoclastogenesis (50 µg/mL) [107]. VitC (12.5 µg/mL) combined with dexamethasone promotes mESC differentiation to adipocytes [108]. In this context, all these differentiations rely on the induction of collagen synthesis. Of note, VitC promotes collagen hydroxylation/maturation and deposition by enhancing the activity of the αKG /Fe^+2^-dependent dioxygenases, P4h.

## 8. Conclusions and Future Perspectives

Increasing evidence points to a strong link between metabolism and epigenetic alteration in cell fate decision. Indeed, many chromatin-modifying enzymes are sensitive to fluctuations in the availability of metabolic intermediaries, which are key substrates or cofactors/enhancers of their activity. Changes in their abundance can influence global levels of DNA and histone methylation and acetylation, which may in turn govern gene expression profiles. In particular, a growing interest is focused on how this metabolic–epigenetic axis may be causative or associated with PSC behavior. Naïve to primed transition, the balance between self-renewal and differentiation, and the reprogramming of somatic cells to iPSCs, are typical cell state transition (CST) in which PSCs undergo a metabolic reprogramming. A key example of the metabolic–epigenetic crosstalk has been reported for one-carbon metabolism, which controls histone 3 methylation levels, a crucial mark for the establishment and definition of different cellular states [23,41,48,109,110,111]. However, how such a global epigenetic modification affects gene expression profiles from a global point of view is unknown and requires further investigation.

It is important to underlie that expression analysis of metabolic genes or the systemic analysis of metabolites (metabolomics) do not always reflect the complexity of the flux of the metabolic reactions, but simply provide a snapshot of the metabolic status of a cell [12]. Moreover, the metabolic profile strictly depends on the culture conditions used to capture or induce in vitro a specific cell state. Indeed, media formulations are often complex and/or not specifically declared. For example, they usually include serum, knockout serum (KOSR), vitamins, aminoacids and other metabolites, whose levels are in some cases not physiological. Thus, the definition of the culture conditions, as well as the proper characterization of the specific cell state analyzed, is crucial when investigating the metabolic regulation of the epigenetic status in CST. In particular, this becomes critical when comparing mouse and human PSCs. Indeed, while for mESCs the culture conditions used to induce the naïve and primed states are well defined, for the human counterparts this is still not completely known. Metabolic and epigenetic alteration must be well interpreted when studying the transition of somatic cells to iPSCs during reprogramming. Indeed, the quality of the iPSCs, i.e., “good” or “bad” iPSCs, is critical in the definition of the metabolic and epigenetic profiles. Additionally, PSC differentiation often results in heterogeneous population and may thus generate confusion when analyzing the metabolic and epigenetic profiles. These important aspects must be taken into account since they may, at least in part, explain some discrepancies observed when comparing the effect of a metabolite during CST. The metabolic-epigenetic crosstalk may also govern cell fate choice in other CST, associated with pathologies. Indeed, in the context of cancer progression in which cells acquire invasive and malignant phenotypes, micronutrients and metabolites availability in the tumor microenvironment may influence the epigenetic landscape, and thus the behavior of cancer cells [112]. 

**Figure 5:**
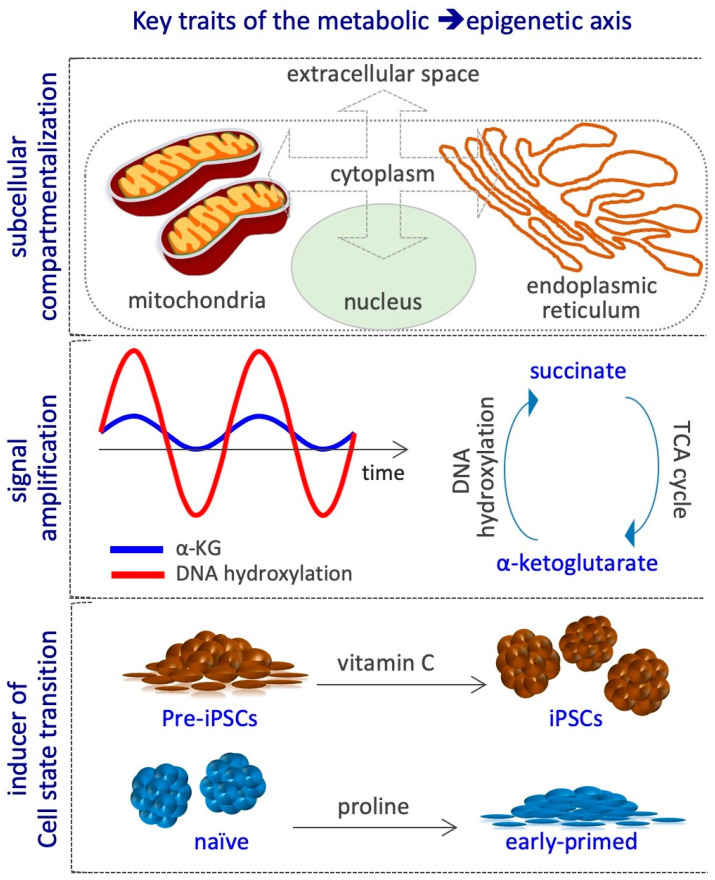
Key traits of the metabolic–epigenetic axis. Scheme representing metabolites subcellular compartmentalization; an example of signal amplification, i.e., TCA cycle regenerate αKG, which is consumed (converted into succinate) during DNA hydroxylation (demethylation); VitC and Proline as examples of metabolites that directly influence CST.

At least three key aspects define the metabolic–epigenetic crosstalk. First, it involves different cellular compartments, e.g., extracellular space, cytoplasm, mitochondria, endoplasmic reticulum and nucleus, and thus requires specific metabolites transporters/carriers, which are not completely known. Indeed, variations in metabolites concentration may occur in specific subcellular compartments during CST, without altering the global cellular levels (Figure 5). Yet, the methodologies available so far to measure metabolites levels within each compartment are poorly developed, thus limiting the possibility to investigate this critical aspect. Second, the metabolic cycles, such as that of folate, methionine, and TCA may continuously regenerate the active form of the substrates and cofactors of the epigenetic enzymes, thus amplifying their signals without the need of constant exogenous supplementation (Figure 5). However, different studies show that although the cells are able to endogenously synthetize some of these metabolites, including acetyl-CoA, L-Pro, αKG and VitC, their exogenous supplementation largely impact on epigenetic alteration, thus suggesting that their levels are limiting for epigenetic reactions in that specific context [20,67,72,75,76,77,78,83,91,93]. The mechanisms by which cells maintain low levels of epigenetic metabolites are poorly investigated, except for the non-essential amino acid l-Pro. Indeed, an autoregulatory loop between the amino acid starvation pathway (AAR/Atf4) and the genes coding for l-Pro biosynthesis enzymes (*Aldh18a1* and *Pycr1*) maintains l-Pro levels limiting to preserve naïve ESC identity [71]. Third, CST is usually induced by specific growth factors and cytokines, and the transition is associated with metabolic and epigenetic reprogramming. For example, Myc controls hPSC fate decisions by regulating the transcription of metabolic genes, in particular up-regulating glycolytic enzymes, which are essential for the establishment of pluripotency [8,113,114]. Hypoxia-inducible factor 1α (HIF1α) induces ESCs towards a metabolic switch from bivalent to a glycolytic Activin/Nodal-dependent primed state, and is also required during the early stages of reprogramming [5,115]. Cripto/Smad2 controls the metabolic reprogramming occurring during naïve to primed transition [116]. Stat3, acting downstream of LIF, increases the expression of mitochondrial-encoded genes, enhancing OX/PHOS metabolism [117]. Zic3 and Esrrb transcription factors finely modulate the expression of glycolytic and OX/PHOS genes, inducing naïve pluripotency and enhancing somatic cell reprogramming [118,119]. LIN28 binds to and regulates key OX/PHOS proteins, maintaining a low mitochondrial metabolism associated with the primed state [120]. Conversely, the metabolic state can also modulate the activity of transcription factors involved in CST, as shown for the oxidation of key cysteine residues of Oct4 in the absence of glutamine [121]. Whether metabolic reprogramming precedes or is rather a consequence of the CST and the epigenetic modifications is not clear. Few examples exist where the availability of a metabolite is able per sè to induce CST and the associated epigenetic alterations; for instance, i) VitC supplementation induces the pre-iPSCs to iPSCs transition [81]; ii) exogenously supplied l-Pro force ESCs to exit the naïve state and to acquire an early-primed state of pluripotency [72] (Figure 5).

Extensive metabolic changes occur during the early phases of development. In particular oxygen consumption increases in the blastocyst stage and decreases by day E6.5 after implantation, stage in which glucose is the main metabolite consumed. This metabolic switch is critical for proper embryo development and is well reflected in PSCs in vitro naïve and primed counterparts [122,123,124]. Moreover, it has been reported that some mitochondrial TCA cycle enzymes translocate into the nucleus and are critical for zygote genome activation [125]. How metabolite availability impacts on chromatin modifications during the pre/early-post implantation development is an emerging field of interest. However, exploring this link may be complex, especially due to the limitations of current technologies, such as metabolic flux analysis, which may be challenging when applied to few cells as in early embryogenesis [126]. Embryos deficient in Myc resemble diapause embryos and show naïve features, including lower expression of glycolytic genes [127]. Inhibition of mTOR in blastocyst led to paused embryos, with global transcriptome repression and reduction of histone modifications associated with gene activity [128]. However, the link between transcription factor-mediated metabolic changes and epigenetic modifications deserves further investigation.

Finally, other classes of enzymes, which require the same substrates and cofactors, are involved in the methylation of RNA, which has been also correlated to PSC transition, e.g., naïve to primed [129,130,131,132]. In particular, 6-methyl-adenosine (6-mA), which is deposited by METTL3/METTL14 complex, requires SAM. This modification is removed by the VitC/ αKG /Fe^+2^-dependent dioxygenases FTO and ALKBH5, which similarly to TET and JMJ depend on αKG and VitC availability. Their activity is crucial for RNA metabolism and thus is strictly associated with transcriptome changes that may underlie CST. However, the connection between metabolism and RNA methylation is still poorly investigated, and will deserve further investigation.

In conclusion, despite our understanding of metabolic and epigenetic crosstalk in CST still being incomplete, and although the development of new technologies to better characterize the cellular metabolic status as well as the right definition of the cell culture conditions are fundamental, our knowledge in the field has rapidly improved.

## References

[1] Tatapudy S., Aloisio F., Barber D., Nystul T. (2017). Cell fate decisions: Emerging roles for metabolic signals and cell morphology. EMBO Rep..

[2] Reid M.A., Dai Z., Locasale J.W. (2017). The impact of cellular metabolism on chromatin dynamics and epigenetics. Nat. Cell Biol..

[3] Takahashi K., Yamanaka S. (2006). Induction of pluripotent stem cells from mouse embryonic and adult fibroblast cultures by defined factors. Cell.

[4] Takahashi K., Tanabe K., Ohnuki M., Narita M., Ichisaka T., Tomoda K., Yamanaka S. (2007). Induction of pluripotent stem cells from adult human fibroblasts by defined factors. Cell.

[5] Zhou W., Choi M., Margineantu D., Margaretha L., Hesson J., Cavanaugh C., Blau C.A., Horwitz M.S., Hockenbery D., Ware C. (2012). HIF1alpha induced switch from bivalent to exclusively glycolytic metabolism during ESC-to-EpiSC/hESC transition. EMBO J..

[6] Gu W., Gaeta X., Sahakyan A., Chan A.B., Hong C.S., Kim R., Braas D., Plath K., Lowry W.E., Christofk H.R. (2016). Glycolytic Metabolism Plays a Functional Role in Regulating Human Pluripotent Stem Cell State. Cell Stem Cell.

[7] Varum S., Rodrigues A.S., Moura M.B., Momcilovic O., Easley C.A.t., Ramalho-Santos J., Van Houten B., Schatten G. (2011). Energy metabolism in human pluripotent stem cells and their differentiated counterparts. PLoS One.

[8] Cliff T.S., Wu T., Boward B.R., Yin A., Yin H., Glushka J.N., Prestegaard J.H., Dalton S. (2017). MYC Controls Human Pluripotent Stem Cell Fate Decisions through Regulation of Metabolic Flux. Cell Stem Cell.

[9] Folmes C.D., Nelson T.J., Martinez-Fernandez A., Arrell D.K., Lindor J.Z., Dzeja P.P., Ikeda Y., Perez-Terzic C., Terzic A. (2011). Somatic oxidative bioenergetics transitions into pluripotency-dependent glycolysis to facilitate nuclear reprogramming. Cell Metab..

[10] Panopoulos A.D., Yanes O., Ruiz S., Kida Y.S., Diep D., Tautenhahn R., Herrerias A., Batchelder E.M., Plongthongkum N., Lutz M. (2012). The metabolome of induced pluripotent stem cells reveals metabolic changes occurring in somatic cell reprogramming. Cell Res..

[11] Zhang J., Nuebel E., Daley G.Q., Koehler C.M., Teitell M.A. (2012). Metabolic regulation in pluripotent stem cells during reprogramming and self-renewal. Cell Stem Cell.

[12] Chandrasekaran S., Zhang J., Sun Z., Zhang L., Ross C.A., Huang Y.C., Asara J.M., Li H., Daley G.Q., Collins J.J. (2017). Comprehensive Mapping of Pluripotent Stem Cell Metabolism Using Dynamic Genome-Scale Network Modeling. Cell Rep..

[13] Ryall J.G., Cliff T., Dalton S., Sartorelli V. (2015). Metabolic Reprogramming of Stem Cell Epigenetics. Cell Stem Cell.

[14] Sivanand S., Viney I., Wellen K.E. (2018). Spatiotemporal Control of Acetyl-CoA Metabolism in Chromatin Regulation. Trends Biochem. Sci..

[15] Ye C., Tu B.P. (2018). Sink into the Epigenome: Histones as Repositories That Influence Cellular Metabolism. Trends Endocrinol. Metab..

[16] Cai L., Sutter B.M., Li B., Tu B.P. (2011). Acetyl-CoA induces cell growth and proliferation by promoting the acetylation of histones at growth genes. Mol. Cell.

[17] Yanes O., Clark J., Wong D.M., Patti G.J., Sanchez-Ruiz A., Benton H.P., Trauger S.A., Desponts C., Ding S., Siuzdak G. (2010). Metabolic oxidation regulates embryonic stem cell differentiation. Nat. Chem. Biol..

[18] Wang L., Zhang T., Wang L., Cai Y., Zhong X., He X., Hu L., Tian S., Wu M., Hui L. (2017). Fatty acid synthesis is critical for stem cell pluripotency via promoting mitochondrial fission. EMBO J..

[19] Shyh-Chang N., Daley G.Q. (2015). Metabolic switches linked to pluripotency and embryonic stem cell differentiation. Cell Metab..

[20] Moussaieff A., Rouleau M., Kitsberg D., Cohen M., Levy G., Barasch D., Nemirovski A., Shen-Orr S., Laevsky I., Amit M. (2015). Glycolysis-mediated changes in acetyl-CoA and histone acetylation control the early differentiation of embryonic stem cells. Cell Metab..

[21] Miller S.J. (2004). Cellular and physiological effects of short-chain fatty acids. Mini Rev. Med. Chem.

[22] Mali P., Chou B.K., Yen J., Ye Z., Zou J., Dowey S., Brodsky R.A., Ohm J.E., Yu W., Baylin S.B. (2010). Butyrate greatly enhances derivation of human induced pluripotent stem cells by promoting epigenetic remodeling and the expression of pluripotency-associated genes. Stem Cells.

[23] Shyh-Chang N., Locasale J.W., Lyssiotis C.A., Zheng Y., Teo R.Y., Ratanasirintrawoot S., Zhang J., Onder T., Unternaehrer J.J., Zhu H. (2012). Influence of Threonine Metabolism on S-Adenosylmethionine and Histone Methylation. Science.

[24] Feil R., Fraga M.F. (2012). Epigenetics and the environment: Emerging patterns and implications. Nat. Rev. Genet..

[25] Fang Y., Tang S., Li X. (2019). Sirtuins in Metabolic and Epigenetic Regulation of Stem Cells. Trends Endocrinol. Metab..

[26] Calvanese V., Lara E., Suarez-Alvarez B., Abu Dawud R., Vazquez-Chantada M., Martinez-Chantar M.L., Embade N., Lopez-Nieva P., Horrillo A., Hmadcha A. (2010). Sirtuin 1 regulation of developmental genes during differentiation of stem cells. Proc. Natl. Acad. Sci. USA.

[27] Tang S., Huang G., Fan W., Chen Y., Ward J.M., Xu X., Xu Q., Kang A., McBurney M.W., Fargo D.C. (2014). SIRT1-mediated deacetylation of CRABPII regulates cellular retinoic acid signaling and modulates embryonic stem cell differentiation. Mol Cell.

[28] Williams E.O., Taylor A.K., Bell E.L., Lim R., Kim D.M., Guarente L. (2016). Sirtuin 1 Promotes Deacetylation of Oct4 and Maintenance of Naive Pluripotency. Cell Rep..

[29] Heo J., Lim J., Lee S., Jeong J., Kang H., Kim Y., Kang J.W., Yu H.Y., Jeong E.M., Kim K. (2017). Sirt1 Regulates DNA Methylation and Differentiation Potential of Embryonic Stem Cells by Antagonizing Dnmt3l. Cell Rep..

[30] Xu P., Wang T.T., Liu X.Z., Wang N.Y., Sun L.H., Zhang Z.Q., Chen H.Z., Lv X., Huang Y., Liu D.P. (2019). Sirt6 regulates efficiency of mouse somatic reprogramming and maintenance of pluripotency. Stem Cell Res..

[31] Etchegaray J.P., Chavez L., Huang Y., Ross K.N., Choi J., Martinez-Pastor B., Walsh R.M., Sommer C.A., Lienhard M., Gladden A. (2015). The histone deacetylase SIRT6 controls embryonic stem cell fate via TET-mediated production of 5-hydroxymethylcytosine. Nat. Cell Biol..

[32] Son M.J., Son M.Y., Seol B., Kim M.J., Yoo C.H., Han M.K., Cho Y.S. (2013). Nicotinamide overcomes pluripotency deficits and reprogramming barriers. Stem Cells.

[33] Meng Y., Ren Z., Xu F., Zhou X., Song C., Wang V.Y., Liu W., Lu L., Thomson J.A., Chen G. (2018). Nicotinamide Promotes Cell Survival and Differentiation as Kinase Inhibitor in Human Pluripotent Stem Cells. Stem Cell Rep..

[34] Kropp E.M., Oleson B.J., Broniowska K.A., Bhattacharya S., Chadwick A.C., Diers A.R., Hu Q., Sahoo D., Hogg N., Boheler K.R. (2015). Inhibition of an NAD(+) salvage pathway provides efficient and selective toxicity to human pluripotent stem cells. Stem Cells Transl. Med..

[35] Griffin S.M., Pickard M.R., Orme R.P., Hawkins C.P., Williams A.C., Fricker R.A. (2017). Nicotinamide alone accelerates the conversion of mouse embryonic stem cells into mature neuronal populations. PLoS One.

[36] Buchholz D.E., Pennington B.O., Croze R.H., Hinman C.R., Coffey P.J., Clegg D.O. (2013). Rapid and efficient directed differentiation of human pluripotent stem cells into retinal pigmented epithelium. Stem Cells Transl. Med..

[37] Nostro M.C., Sarangi F., Yang C., Holland A., Elefanty A.G., Stanley E.G., Greiner D.L., Keller G. (2015). Efficient generation of NKX6-1+ pancreatic progenitors from multiple human pluripotent stem cell lines. Stem Cell Rep..

[38] Idelson M., Alper R., Obolensky A., Ben-Shushan E., Hemo I., Yachimovich-Cohen N., Khaner H., Smith Y., Wiser O., Gropp M. (2009). Directed differentiation of human embryonic stem cells into functional retinal pigment epithelium cells. Cell Stem Cell.

[39] Parsons X.H., Teng Y.D., Parsons J.F., Snyder E.Y., Smotrich D.B., Moore D.A. (2011). Efficient derivation of human cardiac precursors and cardiomyocytes from pluripotent human embryonic stem cells with small molecule induction. J. Vis. Exp.

[40] Vaca P., Berna G., Araujo R., Carneiro E.M., Bedoya F.J., Soria B., Martin F. (2008). Nicotinamide induces differentiation of embryonic stem cells into insulin-secreting cells. Exp. Cell Res..

[41] Mentch S.J., Locasale J.W. (2016). One-carbon metabolism and epigenetics: Understanding the specificity. Ann. N. Y. Acad. Sci..

[42] Yan Q., Xu J., Hu W., Li Z., Wu J., Zhang S. (2014). Transient folate deprivation facilitates the generation of mouse-induced pluripotent stem cells. Cell Biol. Int..

[43] Hu W.T., Yan Q.Y., Fang Y., Qiu Z.D., Zhang S.M. (2014). Transient folate deprivation in combination with small-molecule compounds facilitates the generation of somatic cell-derived pluripotent stem cells in mice. J. Huazhong Univ. Sci. Technol. Med. Sci.

[44] Kasulanati S., Venkatesan V. (2018). Understanding pluripotency under folic acid deficiency using embryonic stem cells as an in vitro model. Med. Hypotheses.

[45] Chang S., Wang L., Guan Y., Shangguan S., Du Q., Wang Y., Zhang T., Zhang Y. (2013). Long interspersed nucleotide element-1 hypomethylation in folate-deficient mouse embryonic stem cells. J. Cell Biochem..

[46] Wei T., Jia W., Qian Z., Zhao L., Yu Y., Li L., Wang C., Zhang W., Liu Q., Yang D. (2017). Folic Acid Supports Pluripotency and Reprogramming by Regulating LIF/STAT3 and MAPK/ERK Signaling. Stem Cells Dev..

[47] Liang Y., Li Y., Li Z., Liu Z., Zhang Z., Chang S., Wu J. (2012). Mechanism of folate deficiency-induced apoptosis in mouse embryonic stem cells: Cell cycle arrest/apoptosis in G1/G0 mediated by microRNA-302a and tumor suppressor gene Lats2. Int. J. Biochem Cell Biol..

[48] Fawal M.A., Jungas T., Kischel A., Audouard C., Iacovoni J.S., Davy A. (2018). Cross Talk between One-Carbon Metabolism, Eph Signaling, and Histone Methylation Promotes Neural Stem Cell Differentiation. Cell Rep..

[49] Valensisi C., Andrus C., Buckberry S., Doni Jayavelu N., Lund R.J., Lister R., Hawkins R.D. (2017). Epigenomic Landscapes of hESC-Derived Neural Rosettes: Modeling Neural Tube Formation and Diseases. Cell Rep..

[50] Chen Y., Wang Z., Xie Y., Guo X., Tang X., Wang S., Yang S., Chen K., Niu Y., Ji W. (2012). Folic acid deficiency inhibits neural rosette formation and neuronal differentiation from rhesus monkey embryonic stem cells. J. Neurosci. Res..

[51] Sahakyan V., Duelen R., Tam W.L., Roberts S.J., Grosemans H., Berckmans P., Ceccarelli G., Pelizzo G., Broccoli V., Deprest J. (2018). Folic Acid Exposure Rescues Spina Bifida Aperta Phenotypes in Human Induced Pluripotent Stem Cell Model. Sci. Rep..

[52] Kilberg M.S., Terada N., Shan J. (2016). Influence of Amino Acid Metabolism on Embryonic Stem Cell Function and Differentiation. Adv. Nutr..

[53] Wang J., Alexander P., Wu L., Hammer R., Cleaver O., McKnight S.L. (2009). Dependence of mouse embryonic stem cells on threonine catabolism. Science.

[54] Alexander P.B., Wang J., McKnight S.L. (2011). Targeted killing of a mammalian cell based upon its specialized metabolic state. Proc. Natl. Acad. Sci. USA.

[55] Han C., Gu H., Wang J., Lu W., Mei Y., Wu M. (2013). Regulation of L-threonine dehydrogenase in somatic cell reprogramming. Stem Cells.

[56] Chen G., Wang J. (2019). A regulatory circuitry locking pluripotent stemness to embryonic stem cell: Interaction between threonine catabolism and histone methylation. Semin. Cancer Biol..

[57] Shiraki N., Shiraki Y., Tsuyama T., Obata F., Miura M., Nagae G., Aburatani H., Kume K., Endo F., Kume S. (2014). Methionine metabolism regulates maintenance and differentiation of human pluripotent stem cells. Cell Metab..

[58] Fernandez-Arroyo S., Cuyas E., Bosch-Barrera J., Alarcon T., Joven J., Menendez J.A. (2015). Activation of the methylation cycle in cells reprogrammed into a stem cell-like state. Oncoscience.

[59] Tang S., Fang Y., Huang G., Xu X., Padilla-Banks E., Fan W., Xu Q., Sanderson S.M., Foley J.F., Dowdy S. (2017). Methionine metabolism is essential for SIRT1-regulated mouse embryonic stem cell maintenance and embryonic development. EMBO J..

[60] Chan Y.S., Goke J., Ng J.H., Lu X., Gonzales K.A., Tan C.P., Tng W.Q., Hong Z.Z., Lim Y.S., Ng H.H. (2013). Induction of a human pluripotent state with distinct regulatory circuitry that resembles preimplantation epiblast. Cell Stem Cell.

[61] Duggal G., Warrier S., Ghimire S., Broekaert D., Van der Jeught M., Lierman S., Deroo T., Peelman L., Van Soom A., Cornelissen R. (2015). Alternative Routes to Induce Naive Pluripotency in Human Embryonic Stem Cells. Stem Cells.

[62] Gafni O., Weinberger L., Mansour A.A., Manor Y.S., Chomsky E., Ben-Yosef D., Kalma Y., Viukov S., Maza I., Zviran A. (2013). Derivation of novel human ground state naive pluripotent stem cells. Nature.

[63] Ware C.B., Nelson A.M., Mecham B., Hesson J., Zhou W., Jonlin E.C., Jimenez-Caliani A.J., Deng X., Cavanaugh C., Cook S. (2014). Derivation of naive human embryonic stem cells. Proc. Natl. Acad. Sci. USA.

[64] Takashima Y., Guo G., Loos R., Nichols J., Ficz G., Krueger F., Oxley D., Santos F., Clarke J., Mansfield W. (2014). Resetting transcription factor control circuitry toward ground-state pluripotency in human. Cell.

[65] Sperber H., Mathieu J., Wang Y., Ferreccio A., Hesson J., Xu Z., Fischer K.A., Devi A., Detraux D., Gu H. (2015). The metabolome regulates the epigenetic landscape during naive-to-primed human embryonic stem cell transition. Nat. Cell Biol..

[66] Bernstein B.E., Mikkelsen T.S., Xie X., Kamal M., Huebert D.J., Cuff J., Fry B., Meissner A., Wernig M., Plath K. (2006). A bivalent chromatin structure marks key developmental genes in embryonic stem cells. Cell.

[67] Comes S., Gagliardi M., Laprano N., Fico A., Cimmino A., Palamidessi A., De Cesare D., De Falco S., Angelini C., Scita G. (2013). L-Proline induces a mesenchymal-like invasive program in embryonic stem cells by remodeling H3K9 and H3K36 methylation. Stem Cell Rep..

[68] D’Aniello C., Cermola F., Patriarca E.J., Minchiotti G. (2017). Vitamin C in Stem Cell Biology: Impact on Extracellular Matrix Homeostasis and Epigenetics. Stem Cells Int..

[69] Casalino L., Comes S., Lambazzi G., De Stefano B., Filosa S., De Falco S., De Cesare D., Minchiotti G., Patriarca E.J. (2011). Control of embryonic stem cell metastability by L-proline catabolism. J. Mol. Cell Biol..

[70] Washington J.M., Rathjen J., Felquer F., Lonic A., Bettess M.D., Hamra N., Semendric L., Tan B.S., Lake J.A., Keough R.A. (2010). L-Proline induces differentiation of ES cells: A novel role for an amino acid in the regulation of pluripotent cells in culture. Am. J. Physiol. Cell Physiol..

[71] D’Aniello C., Fico A., Casalino L., Guardiola O., Di Napoli G., Cermola F., De Cesare D., Tate R., Cobellis G., Patriarca E.J. (2015). A novel autoregulatory loop between the Gcn2-Atf4 pathway and (L)-Proline [corrected] metabolism controls stem cell identity. Cell Death Differ..

[72] D’Aniello C., Habibi E., Cermola F., Paris D., Russo F., Fiorenzano A., Di Napoli G., Melck D.J., Cobellis G., Angelini C. (2017). Vitamin C and l-Proline Antagonistic Effects Capture Alternative States in the Pluripotency Continuum. Stem Cell Rep..

[73] D’Aniello C., Cermola F., Palamidessi A., Wanderlingh L.G., Gagliardi M., Migliaccio A., Varrone F., Casalino L., Matarazzo M.R., De Cesare D. (2019). Collagen prolyl hydroxylation-dependent metabolic perturbation governs epigenetic remodeling and mesenchymal transition in pluripotent and cancer cells. Cancer Res..

[74] Ohnishi Y., Huber W., Tsumura A., Kang M., Xenopoulos P., Kurimoto K., Oles A.K., Arauzo-Bravo M.J., Saitou M., Hadjantonakis A.K. (2014). Cell-to-cell expression variability followed by signal reinforcement progressively segregates early mouse lineages. Nat. Cell Biol..

[75] Carey B.W., Finley L.W., Cross J.R., Allis C.D., Thompson C.B. (2015). Intracellular alpha-ketoglutarate maintains the pluripotency of embryonic stem cells. Nature.

[76] Hwang I.Y., Kwak S., Lee S., Kim H., Lee S.E., Kim J.H., Kim Y.A., Jeon Y.K., Chung D.H., Jin X. (2016). Psat1-Dependent Fluctuations in alpha-Ketoglutarate Affect the Timing of ESC Differentiation. Cell Metab..

[77] Tischler J., Gruhn W.H., Reid J., Allgeyer E., Buettner F., Marr C., Theis F., Simons B.D., Wernisch L., Surani M.A. (2019). Metabolic regulation of pluripotency and germ cell fate through alpha-ketoglutarate. EMBO J..

[78] TeSlaa T., Chaikovsky A.C., Lipchina I., Escobar S.L., Hochedlinger K., Huang J., Graeber T.G., Braas D., Teitell M.A. (2016). alpha-Ketoglutarate Accelerates the Initial Differentiation of Primed Human Pluripotent Stem Cells. Cell Metab..

[79] Zhang Z., He C., Zhang L., Zhu T., Lv D., Li G., Song Y., Wang J., Wu H., Ji P. (2019). Alpha-ketoglutarate affects murine embryo development through metabolic and epigenetic modulations. Reproduction.

[80] Cimmino L., Neel B.G., Aifantis I. (2018). Vitamin C in Stem Cell Reprogramming and Cancer. Trends Cell Biol..

[81] Esteban M.A., Wang T., Qin B., Yang J., Qin D., Cai J., Li W., Weng Z., Chen J., Ni S. (2010). Vitamin C enhances the generation of mouse and human induced pluripotent stem cells. Cell Stem Cell.

[82] Esteban M.A., Pei D. (2012). Vitamin C improves the quality of somatic cell reprogramming. Nat. Genet..

[83] Wang T., Chen K., Zeng X., Yang J., Wu Y., Shi X., Qin B., Zeng L., Esteban M.A., Pan G. (2011). The histone demethylases Jhdm1a/1b enhance somatic cell reprogramming in a vitamin-C-dependent manner. Cell Stem Cell.

[84] Chen J., Liu H., Liu J., Qi J., Wei B., Yang J., Liang H., Chen Y., Chen J., Wu Y. (2013). H3K9 methylation is a barrier during somatic cell reprogramming into iPSCs. Nat. Genet..

[85] Stadtfeld M., Apostolou E., Ferrari F., Choi J., Walsh R.M., Chen T., Ooi S.S., Kim S.Y., Bestor T.H., Shioda T. (2012). Ascorbic acid prevents loss of Dlk1-Dio3 imprinting and facilitates generation of all-iPS cell mice from terminally differentiated B cells. Nat. Genet..

[86] Xu X., Smorag L., Nakamura T., Kimura T., Dressel R., Fitzner A., Tan X., Linke M., Zechner U., Engel W. (2015). Dppa3 expression is critical for generation of fully reprogrammed iPS cells and maintenance of Dlk1-Dio3 imprinting. Nat. Commun.

[87] Gao Y., Han Z., Li Q., Wu Y., Shi X., Ai Z., Du J., Li W., Guo Z., Zhang Y. (2015). Vitamin C induces a pluripotent state in mouse embryonic stem cells by modulating microRNA expression. Febs. J..

[88] Doege C.A., Inoue K., Yamashita T., Rhee D.B., Travis S., Fujita R., Guarnieri P., Bhagat G., Vanti W.B., Shih A. (2012). Early-stage epigenetic modification during somatic cell reprogramming by Parp1 and Tet2. Nature.

[89] Chen J., Guo L., Zhang L., Wu H., Yang J., Liu H., Wang X., Hu X., Gu T., Zhou Z. (2013). Vitamin C modulates TET1 function during somatic cell reprogramming. Nat. Genet..

[90] Gao Y., Chen J., Li K., Wu T., Huang B., Liu W., Kou X., Zhang Y., Huang H., Jiang Y. (2013). Replacement of Oct4 by Tet1 during iPSC induction reveals an important role of DNA methylation and hydroxymethylation in reprogramming. Cell Stem Cell.

[91] Blaschke K., Ebata K.T., Karimi M.M., Zepeda-Martinez J.A., Goyal P., Mahapatra S., Tam A., Laird D.J., Hirst M., Rao A. (2013). Vitamin C induces Tet-dependent DNA demethylation and a blastocyst-like state in ES cells. Nature.

[92] Gao Y., Yang L., Chen L., Wang X., Wu H., Ai Z., Du J., Liu Y., Shi X., Wu Y. (2013). Vitamin C facilitates pluripotent stem cell maintenance by promoting pluripotency gene transcription. Biochimie.

[93] Chung T.L., Brena R.M., Kolle G., Grimmond S.M., Berman B.P., Laird P.W., Pera M.F., Wolvetang E.J. (2010). Vitamin C promotes widespread yet specific DNA demethylation of the epigenome in human embryonic stem cells. Stem Cells.

[94] Hore T.A., von Meyenn F., Ravichandran M., Bachman M., Ficz G., Oxley D., Santos F., Balasubramanian S., Jurkowski T.P., Reik W. (2016). Retinol and ascorbate drive erasure of epigenetic memory and enhance reprogramming to naive pluripotency by complementary mechanisms. Proc. Natl. Acad. Sci. USA.

[95] Walter M., Teissandier A., Perez-Palacios R., Bourc’his D. (2016). An epigenetic switch ensures transposon repression upon dynamic loss of DNA methylation in embryonic stem cells. Elife.

[96] Dawlaty M.M., Breiling A., Le T., Barrasa M.I., Raddatz G., Gao Q., Powell B.E., Cheng A.W., Faull K.F., Lyko F. (2014). Loss of Tet enzymes compromises proper differentiation of embryonic stem cells. Dev Cell.

[97] Langlois T., da Costa Reis Monte-Mor B., Lenglet G., Droin N., Marty C., Le Couedic J.P., Almire C., Auger N., Mercher T., Delhommeau F. (2014). TET2 deficiency inhibits mesoderm and hematopoietic differentiation in human embryonic stem cells. Stem Cells.

[98] Norman L., Tarrant P., Chevassut T. (2014). TET2 Inhibits Differentiation of Embryonic Stem Cells but Does Not Overcome Methylation-Induced Gene Silencing. Bone Marrow Res..

[99] Dawlaty M.M., Breiling A., Le T., Raddatz G., Barrasa M.I., Cheng A.W., Gao Q., Powell B.E., Li Z., Xu M. (2013). Combined deficiency of Tet1 and Tet2 causes epigenetic abnormalities but is compatible with postnatal development. Dev Cell.

[100] Koh K.P., Yabuuchi A., Rao S., Huang Y., Cunniff K., Nardone J., Laiho A., Tahiliani M., Sommer C.A., Mostoslavsky G. (2011). Tet1 and Tet2 regulate 5-hydroxymethylcytosine production and cell lineage specification in mouse embryonic stem cells. Cell Stem Cell.

[101] Kang J., Lienhard M., Pastor W.A., Chawla A., Novotny M., Tsagaratou A., Lasken R.S., Thompson E.C., Surani M.A., Koralov S.B. (2015). Simultaneous deletion of the methylcytosine oxidases Tet1 and Tet3 increases transcriptome variability in early embryogenesis. Proc. Natl. Acad. Sci. USA.

[102] Ross S.E., Bogdanovic O. (2019). TET enzymes, DNA demethylation and pluripotency. Biochem Soc Trans..

[103] Takahashi T., Lord B., Schulze P.C., Fryer R.M., Sarang S.S., Gullans S.R., Lee R.T. (2003). Ascorbic acid enhances differentiation of embryonic stem cells into cardiac myocytes. Circulation.

[104] Crescini E., Gualandi L., Uberti D., Prandelli C., Presta M., Dell’Era P. (2013). Ascorbic acid rescues cardiomyocyte development in Fgfr1(-/-) murine embryonic stem cells. Biochim Biophys Acta.

[105] Cao N., Liu Z., Chen Z., Wang J., Chen T., Zhao X., Ma Y., Qin L., Kang J., Wei B. (2012). Ascorbic acid enhances the cardiac differentiation of induced pluripotent stem cells through promoting the proliferation of cardiac progenitor cells. Cell Res..

[106] Buttery L.D., Bourne S., Xynos J.D., Wood H., Hughes F.J., Hughes S.P., Episkopou V., Polak J.M. (2001). Differentiation of osteoblasts and in vitro bone formation from murine embryonic stem cells. Tissue Eng.

[107] Tsuneto M., Yamazaki H., Yoshino M., Yamada T., Hayashi S. (2005). Ascorbic acid promotes osteoclastogenesis from embryonic stem cells. Biochem. Biophys. Res. Commun.

[108] Cuaranta-Monroy I., Simandi Z., Kolostyak Z., Doan-Xuan Q.M., Poliska S., Horvath A., Nagy G., Bacso Z., Nagy L. (2014). Highly efficient differentiation of embryonic stem cells into adipocytes by ascorbic acid. Stem Cell Res..

[109] Garcia B.A., Luka Z., Loukachevitch L.V., Bhanu N.V., Wagner C. (2016). Folate deficiency affects histone methylation. Med. Hypotheses.

[110] Mentch S.J., Mehrmohamadi M., Huang L., Liu X., Gupta D., Mattocks D., Gomez Padilla P., Ables G., Bamman M.M., Thalacker-Mercer A.E. (2015). Histone Methylation Dynamics and Gene Regulation Occur through the Sensing of One-Carbon Metabolism. Cell Metab..

[111] Benayoun B.A., Pollina E.A., Ucar D., Mahmoudi S., Karra K., Wong E.D., Devarajan K., Daugherty A.C., Kundaje A.B., Mancini E. (2014). H3K4me3 breadth is linked to cell identity and transcriptional consistency. Cell.

[112] Mahmoud A.M., Ali M.M. (2019). Methyl Donor Micronutrients that Modify DNA Methylation and Cancer Outcome. Nutrients.

[113] Folmes C.D., Martinez-Fernandez A., Faustino R.S., Yamada S., Perez-Terzic C., Nelson T.J., Terzic A. (2013). Nuclear reprogramming with c-Myc potentiates glycolytic capacity of derived induced pluripotent stem cells. J. Cardiovasc. Transl. Res..

[114] Cao Y., Guo W.T., Tian S., He X., Wang X.W., Liu X., Gu K.L., Ma X., Huang D., Hu L. (2015). miR-290/371-Mbd2-Myc circuit regulates glycolytic metabolism to promote pluripotency. EMBO J..

[115] Mathieu J., Zhou W., Xing Y., Sperber H., Ferreccio A., Agoston Z., Kuppusamy K.T., Moon R.T., Ruohola-Baker H. (2014). Hypoxia-inducible factors have distinct and stage-specific roles during reprogramming of human cells to pluripotency. Cell Stem Cell.

[116] Fiorenzano A., Pascale E., D’Aniello C., Acampora D., Bassalert C., Russo F., Andolfi G., Biffoni M., Francescangeli F., Zeuner A. (2016). Cripto is essential to capture mouse epiblast stem cell and human embryonic stem cell pluripotency. Nat. Commun..

[117] Carbognin E., Betto R.M., Soriano M.E., Smith A.G., Martello G. (2016). Stat3 promotes mitochondrial transcription and oxidative respiration during maintenance and induction of naive pluripotency. EMBO J..

[118] Sone M., Morone N., Nakamura T., Tanaka A., Okita K., Woltjen K., Nakagawa M., Heuser J.E., Yamada Y., Yamanaka S. (2017). Hybrid Cellular Metabolism Coordinated by Zic3 and Esrrb Synergistically Enhances Induction of Naive Pluripotency. Cell Metab..

[119] Kida Y.S., Kawamura T., Wei Z., Sogo T., Jacinto S., Shigeno A., Kushige H., Yoshihara E., Liddle C., Ecker J.R. (2015). ERRs Mediate a Metabolic Switch Required for Somatic Cell Reprogramming to Pluripotency. Cell Stem Cell.

[120] Zhang J., Ratanasirintrawoot S., Chandrasekaran S., Wu Z., Ficarro S.B., Yu C., Ross C.A., Cacchiarelli D., Xia Q., Seligson M. (2016). LIN28 Regulates Stem Cell Metabolism and Conversion to Primed Pluripotency. Cell Stem Cell.

[121] Marsboom G., Zhang G.F., Pohl-Avila N., Zhang Y., Yuan Y., Kang H., Hao B., Brunengraber H., Malik A.B., Rehman J. (2016). Glutamine Metabolism Regulates the Pluripotency Transcription Factor OCT4. Cell Rep..

[122] Houghton F.D., Thompson J.G., Kennedy C.J., Leese H.J. (1996). Oxygen consumption and energy metabolism of the early mouse embryo. Mol. Reprod. Dev..

[123] Mathieu J., Ruohola-Baker H. (2017). Metabolic remodeling during the loss and acquisition of pluripotency. Development.

[124] Boroviak T., Loos R., Lombard P., Okahara J., Behr R., Sasaki E., Nichols J., Smith A., Bertone P. (2015). Lineage-Specific Profiling Delineates the Emergence and Progression of Naive Pluripotency in Mammalian Embryogenesis. Dev. Cell.

[125] Nagaraj R., Sharpley M.S., Chi F., Braas D., Zhou Y., Kim R., Clark A.T., Banerjee U. (2017). Nuclear Localization of Mitochondrial TCA Cycle Enzymes as a Critical Step in Mammalian Zygotic Genome Activation. Cell.

[126] Zhang J., Zhao J., Dahan P., Lu V., Zhang C., Li H., Teitell M.A. (2018). Metabolism in Pluripotent Stem Cells and Early Mammalian Development. Cell Metab..

[127] Scognamiglio R., Cabezas-Wallscheid N., Thier M.C., Altamura S., Reyes A., Prendergast A.M., Baumgartner D., Carnevalli L.S., Atzberger A., Haas S. (2016). Myc Depletion Induces a Pluripotent Dormant State Mimicking Diapause. Cell.

[128] Bulut-Karslioglu A., Biechele S., Jin H., Macrae T.A., Hejna M., Gertsenstein M., Song J.S., Ramalho-Santos M. (2016). Inhibition of mTOR induces a paused pluripotent state. Nature.

[129] Geula S., Moshitch-Moshkovitz S., Dominissini D., Mansour A.A., Kol N., Salmon-Divon M., Hershkovitz V., Peer E., Mor N., Manor Y.S. (2015). Stem cells. m6A mRNA methylation facilitates resolution of naive pluripotency toward differentiation. Science.

[130] Lin S., Gregory R.I. (2014). Methyltransferases modulate RNA stability in embryonic stem cells. Nat. Cell Biol..

[131] Wang Y., Li Y., Toth J.I., Petroski M.D., Zhang Z., Zhao J.C. (2014). N6-methyladenosine modification destabilizes developmental regulators in embryonic stem cells. Nat. Cell Biol..

[132] Batista P.J., Molinie B., Wang J., Qu K., Zhang J., Li L., Bouley D.M., Lujan E., Haddad B., Daneshvar K. (2014). m(6)A RNA modification controls cell fate transition in mammalian embryonic stem cells. Cell Stem Cell.

